# “Why Are You Running Away From Social Media?” Analysis of the Factors Influencing Social Media Fatigue: An Empirical Data Study Based on Chinese Youth

**DOI:** 10.3389/fpsyg.2021.674641

**Published:** 2021-09-21

**Authors:** Yixuan Liu, Jianping He

**Affiliations:** School of Media and Communication, Shenzhen University, Shenzhen, China

**Keywords:** social media, youth group, social media fatigue, influencing factors, social escape

## Abstract

In the digital era, social media is increasingly permeating the fragmented lives of people. While enjoying the convenience and speed of online socializing, people are gradually surrounded by a variety of information. Through observations and interviews, we found that young people are plagued by negative comparisons, interaction dysfunction, information overload, social overload, and intergenerational communication in the process of participating in social media. Increasing numbers of young people feel overwhelmed in the process of online socialization and the phenomenon of social media fatigue (SMF) gradually spreads. This study combined the existing relevant theoretical models of the influencing factors of SMF and the real-life empirical materials of youth groups. Furthermore, this study enriched into new research variables and validated the data of relevant variables through a questionnaire survey (*n* = 663) to explore the influencing factors of SMF of youth groups and inductively analyze the inner logic of the emergence of SMF among such groups. It was found that negative comparison, interaction dysregulation, information overload, social overload, self-efficacy, and impression management all had significant positive effects on SMF behaviors, while privacy anxiety and intergenerational communication had no significant positive effects on SMF behaviors.

## Introduction

The emergence of social media plays an important role in promoting offline social activities and related identity construction (Hercheui, 2011). According to the 47th “statistical report on the development of Internet in China” released by CNNIC, there are more than 900 million Internet users in China, accounting for one-fifth of the Internet users around the world. More and more users share information and establish contacts with others through social media. Among them, young people who are the main users of social media have different degrees of dependence on social media in their daily work and study life and have better psychological and behavioral experience in the use of social media. However, with the explosive development of social media, some “dark side” has gradually emerged in recent years (Salo et al., 2018). Some young people spend most of their time on social media and even indulge in it. However, after immersion and awakening, they become tired of social media. Many users begin to show resistance and fatigue, and the loss of users has gradually become an urgent crisis for each social platform.

As early as 2011, Gartner, a market research organization surveyed 6,295 social media users aged 13–74 in 11 countries and found that the social media market has become mature. Some market segments have shown signs of social fatigue, and 24% of the users now use social media less frequently than when they first used it (Goasduff and Pettey, 2011). In 2018, Hill Holliday released the “Z generation social media survey report,” which shows that more than half of young people have reduced their use of social media, and 34% of young people have stopped accessing social media (Hill Holliday, 2018). When users are surrounded by a lot of information and need to spend a lot of time dealing with these social contacts, some users will choose to stay away from and give up social media. This shows that the activity of social media is declining, and many researchers say that social media fatigue (SMF) may be the direct cause of this phenomenon. At present, the academic circles must calm down, re-examine, and reflect on the relationship between technology and human beings. They must pay attention to the situation of young people as Internet aborigines, bid farewell to alienation, get rid of the control of social media, and explore the way of subject freedom and extrication. Based on this, this study discussed the following issues:

1. Why do young people escape from social media? What are the reasons for escape and fatigue?
2. Which factor has the highest contribution rate in the model? How to explain?

## Literature Review

### Social Media Fatigue

In the academic world, Adam Patrick first noticed that people were inundated by an endless stream of social networking sites. He first proposed the concept of SMF, and then SMF aroused widespread concern. At present, researchers at home and abroad mainly define the concept of SMF from the perspective of emotion and behavior. Some scholars believe that communication media fatigue is a psychological concept, which is the negative emotional response of the audience to social media activities, such as fatigue, boredom, disinterest, and indifference, among others (Ling et al., 2015). Researchers from the perspective of behavior mostly define it as a kind of negative use behavior. Some scholars point out that SMF is not only used to describe depression, exhaustion, and other feelings but also includes low willingness to participate in social media (Bernstein, 2009). The study of Bright and other scholars defines SMF from the perspective of information overload, which is the tendency of the audience to escape from social media when they are exposed to excessive information (Bright et al., 2015).

In addition, relevant studies have proved that when individuals are exposed to information beyond their effective management and scope, they will produce negative emotions and psychological pressure (Cao and Sun, 2018). There are also many studies focusing on the relationship between social fatigue and the psychological state of adolescents. For example, social fatigue is more likely to increase the depression and anxiety of adolescents (Dhir et al., 2018), which will lead to a certain degree of social escape (Zong et al., 2019). In order to get rid of the pressure and negative emotions, some users gradually become numb or turn a blind eye to a large amount of information on social media. This behavior of deliberately avoiding and reducing the use of social media or even giving up the use of social media is called SMF (Bright et al., 2015). It also allows individuals to control, interrupt and switch to social media (Sajad et al., 2018).

### Influencing Factors of SMF

More and more studies have proved that “overload” is usually divided into social overload and information overload, and both have an important impact on SMF (Sasaki et al., 2015). At the same time, technical pressure is also the main reason for the SMF of users (Lee A. R. et al., 2016). Another study on Facebook found that “platform attribute,” “self-immersion,” “information content,” “member interaction,” and “life cycle” of social media may affect social fatigue (Ravindran et al., 2014). In addition, some studies have proved that the daily living environment and personality characteristics of individuals are also important causes of social fatigue (Lee S. B. et al., 2016).

Although previous studies have explained the influence mechanism of overload and technical pressure on SMF in more detail, the main discussion is the negative impact of the influencing factors of social fatigue on the use of social media of users, and less attention is paid to the social interaction attribute and privacy anxiety of social media. At the same time, the task processing of social media mostly exists in young people, who will spend more time on social media or deal with other similar activities (Voorveld and Goot, 2013). They will pay more attention to their impression management and privacy. Through the interview conducted in this study, we found that the privacy anxiety and relational pressure of social media can make young people feel pressure, fatigue, and even anxiety and depression. In order to better understand the phenomenon of SMF of young people, this study explored the SMF of users from the four aspects namely, Relational stress, overload, personal-psychological characteristics, and social behavior characteristics. Furthermore, this study enriched relevant new variable conditions, and further developed and updated the influencing factor model of SMF behavior of young people.

## Research Hypothesis

### Relational Stress (Negative Comparisons, Interaction Dysfunction)

According to the social psychologist Gergen, the whole world is constructed by relationships, our environment is mutually constitutive, and there is no separate self, much less a consciousness that is separated from objects which all means that everything is a product of relationality (Gergen, 2009). The study of Rosa incorporated the ideas of Logan and also emphasized that “self-perceptions and identities are formed from actions, experiences, and relationships, as well as from the space-time, social world, and physical world in which we live.” They further accounted that nowadays, we no longer have the ability to integrate these contexts into our own experiences and actions, we lose control, and we become “ego-depleted” amid overwhelming commitments. Also, we have lost control and are “self-exhausted” amid our own affairs (Rosa, 2013). This “self-exhaustion” is the most pronounced feeling of SMF. In this way, the theoretical perspective of “relational constructs” is naturally linked to SMF as a social theoretical path to explain the problem. This article reflected this relational stress through negative comparisons and interaction dysregulation because interactions can represent relational constructs at the behavioral level and are easier to measure.

In terms of negative comparisons, the study of Swallow found that information posted by others through social media causes users to make correlational comparisons based on their own situations. When users perceive themselves to be at a disadvantage in online social comparisons, they are prone to negative self-evaluations and may even reduce their social media use due to low self-esteem and anxiety (Swallow and Kuiper, 1988).

In terms of interaction dysfunction, when youth groups engage in social media, the online interaction style can make them feel meaningless. Moreover, when faced with cold technological media, they cannot get a sense of the interaction of offline social life. This makes online socializing evoke a feeling of emptiness, intrusiveness, or even negative indifference to them, producing an imbalance in interpersonal interaction (Selwyn, 2003). Online socialization can over-immerse them, leading to a lot of wasted time and out-of-control self-immersion. This interaction dysfunction constructs a theoretical perspective that becomes an explanatory path for the factors influencing SMF. Based on this, this article proposed the following hypotheses.

H1: Negative comparison has a significant positive effect on SMF behavior of youth groups.
H2: Interaction dysregulation has a significant positive effect on SMF behavior of youth groups.

### Overload (Information Overload and Social Overload)

Social media carries a lot of information. Although some information is beneficial to individuals, the receptive and cognitive abilities of people are limited (Lang, 2010). When the amount of information people contact exceeds their ability to accept information, information overload will occur (Eppler and Mengis, 2004). Through interviews and observation, the research team found that in social media with complex information, young people cannot get access to the information they really want to know because they push too much content. Some individuals with low media literacy need to spend a lot of time to identify, which leads to the low efficiency of social media use (Bawden and Robinson, 2009). At the same time, social media platforms usually push much invalid information, such as advertising and false information, among others, which leads to confusion of platform information and negative emotions of individuals.

As an important factor of social fatigue, social overload has been verified in previous studies. Social overload refers to the state in which an individual perceives that he must respond to the excessive social support demands of others on social media (Maier et al., 2015). It also reflects the burden on social media providers caused by the excessive social support needs of others. Some scholars believe that the number of interpersonal relationships that individuals can control at the same time in cognition is about 150. Once the number exceeds the upper limit, there will be no more ability to manage interpersonal relationships (Dunbar, 1992). However, the number of online social interactions on social media has far exceeded this number (Walther et al., 2008). Through interviews and observation, we also found that the interpersonal relationships that young people need to maintain are mainly coming from work, school, kinship, and friends, among others. They need to invest a lot of time to maintain social relations, so they will feel tired. Based on this, this article forwarded the following hypotheses:

H3: Information overload has a significant positive impact on the SMF behavior of youth groups.
H4: Social overload has a significant positive impact on the SMF behavior of youth groups.

### Personal-Psychological Traits (Self-Efficacy and Privacy Anxiety)

Regarding the dimension of psychological characteristics of the youth group, it is divided into two dimensions which measured self-efficacy and privacy anxiety. According to the study of Bandura, self-efficacy refers to the ability and beliefs of a person to organize and perform specific actions (Bandura, 1986). Self-efficacy then has an important role for youth groups during social media use. In a general sense, as social media use increases, the self-efficacy of individuals strengthens. In turn, their expectations of social media use increase, and their experience of engagement further encourages social media use behavior, resulting in a decrease in social fatigue (Eastin and Larose, 2000). When youth groups believe that the skills they have cannot use social media or cannot play various types of social media fluently, they will refuse or reduce the frequency of using social media. Some scholars call this phenomenon as self-efficacy of using social media, which mainly refers to the degree of confidence that users can use the skills they have to complete a certain task. In this regard, self-efficacy affects the behavioral choices, cognitive processes, emotional processes of people, etc. (Huang and Yuan, 2018). Therefore, this study concluded that the more exposure to social media sites, the higher the self-efficacy of individuals and the less likely they are to develop SMF behaviors. Conversely, the lower the self-efficacy, the more likely individuals are to avoid social media and the more likely they are to develop SMF behaviors.

At the same time, in the process of using social media, people will have anxiety because of the privacy exposure problem. Worrying about the privacy exposure problem, they are afraid that the use of social media will expose their own identity information, home address, and phone number, among other salient information. The problem of social media privacy disclosure is mainly due to the existence of some social media platforms “excessive authorization” to disclose user privacy. User information privacy is very attractive to operators, especially when user privacy issues are provided by social media platforms to advertisers or unidentified business, including user identity information confirmation problems (Chen and Michael, 2012). Browsing information disclosure, or privacy disclosure of health status, family status, and work status can infringe personal interests. Some individuals will worry about privacy disclosure and generate anxiety in the process of participating in social media, which leads to social fatigue behavior (Malhotra et al., 2004). Based on this, this article puts forward the following hypotheses:

H5: Self-efficacy has a significant negative impact on the SMF behavior of youth groups.
H6: Privacy anxiety has a significant positive impact on the SMF of youth groups.

### Social Behavior Traits (Impression Management and Interaction Disorder)

Based on previous research and participant observation of SMF in youth groups, this study measured two dimensions of social behavior traits in youth groups namely, individual impression management and intergenerational communication. Individual impression management is a determining factor in social media use, and social media is also a presentation booth for shaping the “idealized” self (Gibbs et al., 2013). The impression management of young people is very important in their online social activities, and they can construct and manage their personal image by posting dynamics. Because they attach more importance to their online social image, this impression management can easily bring a psychological burden to users over time. Impression management theory holds that in order to achieve a certain goal, people may try to manage or change the perception of others to their own impression, and any behavior of individuals or organizations on social media in the network environment may affect their impression. At the same time, scholars also found that in order to create a good image, individuals sometimes have to suppress their emotions and hide their personalities to please others, which makes them feel anxious, tired, and even lose confidence in social media (Jones and Harris, 1967).

In addition, intergenerational communication is also an important variable to be considered in this study. Young people are the natives of the Internet and they are labeled as independent and maverick. They are quick to receive information from the outside world, tend to communicate online, and insist on their own opinions. This can lead to misunderstandings and conflicts. As a result, the intergenerational gap between the two parties is widening, which then affects family relationships. For caring and educational purposes, fathers sometimes take means to interfere with online activities which may reduce the interest of their offspring in social media use (Lee et al., 2018). Based on this, this article proposed the following hypothesis:

H7: impression management has a significant positive impact on the SMF behavior of youth groups.
H8: poor intergenerational communication has a significant positive impact on the SMF behavior of youth groups.

**Figure 1:**
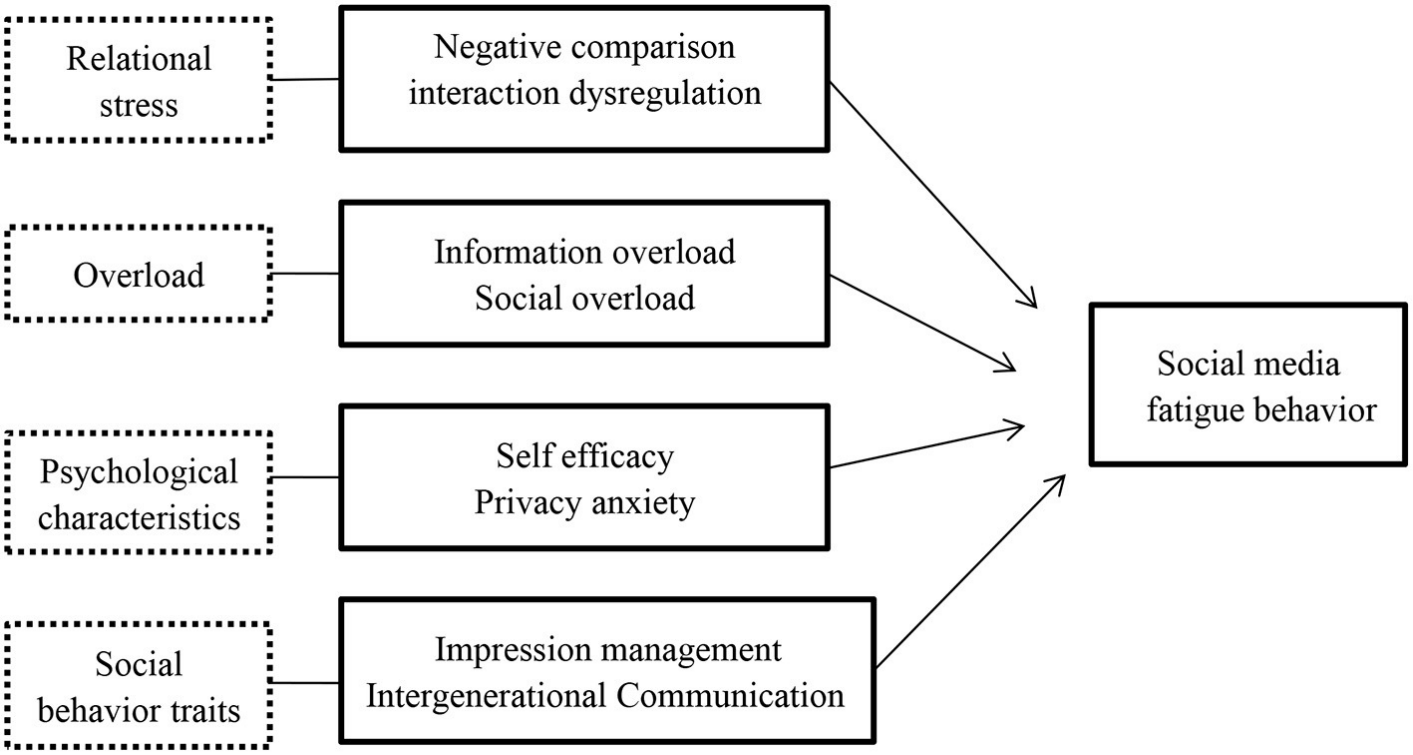
Theoretical model.

Combined with the above assumptions, this article built a research model on the influencing factors of youth SMF behavior, as shown in Figure 1.

## Research Method and Data Collection

The main research method of this study was in-depth interview method and questionnaire method. The research subjects were young social media users aged 18–35 because there is no unified standard for the age definition of the youth group. According to the age definition of youth from the WHO, people aged 18–44 are considered as youth group, and more previous studies also followed this youth group age division standard. Therefore, with reference to previous studies, the subjects selected for this study were consistent with the age range of the youth group. This study did not specify a specific social media for the participants but asked them to choose a social media application that they often use.

First, this study conducted participant observation and in-depth interviews on the social fatigue behaviors of the youth group through qualitative research methods to grasp their daily lifestyles and the composition of their virtual social relationships through the observation of their social fatigue behaviors. However, the observation could only give a general grasp of their SMF behavior, but not a deeper understanding of their psychological motivation. Therefore, this article selected 16 participants with a high willingness to cooperate to conduct semi-structured in-depth interviews. The interviews were conducted online and offline. The research questions were designed according to previous studies. Variables of different dimensions such as relational stress, intergenerational relationships, online identity management, and individual psychology were extracted from the interviews. The interviews also included their growth experiences and the deep psychological motives of SMF behaviors, combining empirical and theoretical materials from shallow to deep.

Next, questionnaires were conducted for the relevant variables to further verify the scientific validity and influence degree of the variables. The questionnaire observation indexes involved questions on a 7-level Likert scale which was set up on a scale of 1–7, with 7 representing “strongly agree,” 4 representing “neutral,” and 1 representing “strongly disagree.” In order to avoid semantic and structural problems of the items and to ensure the convergent and discriminant validity of the items to be measured, 30 masters and doctoral students were invited to conduct a pre-survey of the questionnaire. Furthermore, they were invited to discuss the ambiguities and misunderstandings and to delete and adjust the inappropriate items in the questionnaire.

The survey questionnaire was distributed online in the form of snowball on Weibo, WeChat, QQ community, and friends circle, covering the whole of China. The questionnaires were collected through the professional survey distribution platform “Questionnaire Star” (www.wjx.cn). Participants who agreed to complete the questionnaire were informed of the anonymity of the study. A total of 700 questionnaires were collected, including 500 online questionnaires and 200 offline questionnaires. Thirty-seven unqualified questionnaires were excluded, e.g., unqualified answers, less time spent, inconsistencies, etc., and 663 questionnaires were valid, with a 94% qualification rate. The male sample accounted for 55% and the female sample accounted for 45%. The age of the sample was concentrated between 18 and 23 years old, accounting for 47% of the total number of samples. In the education part, the sample was concentrated on those who have a bachelor's degree, which accounted for 61%. In terms of the frequency of updating personal home page information, the samples focus on publishing information many times a month, accounting for 46%. The basic demographic variables are tabulated as shown in Table 1.

**Table 1:** Statistical table of basic information of effective samples.

**Statistical items**	**Specific content**	**Statistical value**	**Percentage**
Gender	Male	367	55%
	Female	296	45%
Age	18–23	309	47%
	24–29	231	35%
	30–35	123	18%
	Over 35	0	–
Educational	High school	87	13%
background	Undergraduate	402	61%
	Master	153	23%
	Doctor	21	3%
Frequency of	Multiple times per day	71	11%
updating	Multiple times per week	186	28%
dynamics	Multiple times per month	304	46%
	No posting	102	15%

### Variable Measurements and Reliability Testing

The questionnaire involved variables that were measured as much as possible using well-established scales that have been used in related studies, but the questioning of some specific items was fine-tuned to suit the characteristics of the youth group. For the measurement of negative comparisons refer to the scale developed by the study of Gan et al. (2018). For measures of interaction dysregulation, refer to the scale developed by the study of Bright et al. (2015). For measures of social overload, refer to the scale designed by the study of Maier et al. (2015), while for information overload, refer to the scale designed by the study of Koroleva (2010). For measures of self-efficacy and privacy anxiety, refer to the relevant scales in the study of Zhang et al. (2016). The impression management and intergenerational communication scales were selected using the scale designed by the study of Goswami (2012). Lastly, for the measurement of SMF, the main reference was the SMF Scale of Maier et al. (2012).

In this study, IBM SPSS statistics 24 (International Business Machines Corporation, Armonk, New York) was used for data analysis. Reliability analysis was used to calculate the Cronbach alpha coefficient of the scale to judge the stability of the scale. Validity analysis was mainly based on kmo value and Bartlett sphere test. The reliability of the total scale was 0.813, while the reliability of the subscales is higher than 0.7 and most of them are more than 0.8. All these indicated that the internal consistency of the scale in the questionnaire is high and the reliability of the scale is ideal. The kmo value of each variable was above 0.7, and the sig value of the Bartlett sphere test was 0.000, indicating that the validity of each scale was high. The reliability and validity of each scale are shown in Table 2.

**Table 2:** Items and reliability and validity of the scale.

**Variable**	**Code**	**Measuring project**	**Cronbach's Alpha**	**KMO**
Negative comparison	A1	My life is not as exciting as others	0.741	0.750
	A2	Seeing other people's lives increases my anxiety		
	A3	Compared to others, I feel that I am not capable		
Dysfunctional interaction	B1	Most of my social media chats are not for small talk, but for business or other trifles	0.718	0.709
	B2	My friends seldom take the initiative to chat with me every day		
	B3	I spend a lot of time on social media every day		
	B4	I think it's very difficult to control how much time you use social media		
	B5	The overuse of WeChat has affected my normal life, work and study		
Information overload	C1	I'm overwhelmed by the amount of information on social media	0.833	0.713
	C2	Social media is full of useless advertising information		
	C3	Useless information on social media often drowns out important information		
Social overload	D1	I'm very concerned about what's going on in other people's social media	0.804	0.819
	D2	I often keep in touch with others through social media interaction		
	D3	I often express my concern for others by way of likes and comments		
Self-efficacy	E1	I have some expertise information and experience information to share with others in social media	0.841	0.801
	E2	I can view other people's news in social media smoothly, and can comment, reply, forward, like, add or delete comments smoothly		
	E3	I'm sure I can provide valuable information to other people in social media		
Privacy anxiety	F1	I care about my privacy on social media	0.807	0.784
	F2	My personal information is easy to use by advertisers and other marketing personnel		
	F3	I've disclosed a lot of personal privacy information on social media		
Impression management	G1	The content I post on social media is generally positive, healthy and positive	0.753	0.837
	G2	I will carefully select the photos that I am satisfied with and upload them to social media		
	G3	When I make a speech, I think about my own language in time to avoid hurting others		
Intergenerational Communication	H1	My parents usually don't understand me	0.746	0.713
	H2	My elders and I have a big difference in our thinking		
	H3	I don't want my elders to see my social media status		
	H4	There is conflict between my parents and me		
	H5	My parents interfere with my friendships and social activities		
Social media fatigue behavior	I1	I don't use social media to actively chat with others now	0.812	0.705
	I2	Now I have less dynamic comments and praise for others		
	I3	Now my WeChat chat with my friends has become simpler and less in-depth		
	I4	I will often cancel the official account or the push of the screen		
	I5	I don't want to experience the new features of social media anymore		
	I6	I'm going to cancel my current social account		
	I7	I want to go back to the simple and traditional way of social communication (making phone calls, meeting and chatting, etc.)		

## Data Analysis

### Correlation Analysis and Regression Analysis

Using the method of correlation analysis can test the correlation between independent variables and dependent variables. In this study, the independent variables belonged to the fixed distance variables, while the dependent variable was the mean value of the items added under the three dimensions, so it also belonged to the fixed distance variables. Pearson coefficient was used to measure the correlation of variables. Pearson correlation coefficient was used to measure the linear relationship between distance variables. The range of correlation coefficients was between −1 and +1. A positive value represents a positive correlation while a negative value represents a negative correlation. Through correlation analysis, we found that there is a significant correlation between independent variables and dependent variables. The correlation coefficient of the independent variable and dependent variable showed a significant correlation at the level of 0.01 (*P* < 0.01). The specific correlation analysis table is shown in Table 3.

**Table 3:** Correlation analysis table.

**variable**	**Social Media Fatigue**	
Negative comparison	Pearson	0.436^**^
	Sig.	0.000
Interaction dysregulation	Pearson	0.481^*^
	Sig.	0.004
Information overload	Pearson	0.647^**^
	Sig.	0.000
Social overload	Pearson	0.326^**^
	Sig.	0.000
Self-efficacy	Pearson	0.543^*^
	Sig.	0.002
Privacy anxiety	Pearson	0.350^**^
	Sig.	0.000
Impression management	Pearson	0.536^**^
	Sig.	0.000
Intergenerational Communication	Pearson	0.342^**^
	Sig.	0.000

*(N = 663)*.

** *P < 0.01*,

* *P < 0.05*.

The correlations between the variables and factors were verified, and it was confirmed that there was a correlation between all the variables. The next step was performing regression analysis on each variable to further prove whether the research hypothesis is valid. Based on the correlation analysis above, it was found that negative comparison (0.436^**^, *p* < 0.01), interaction dysregulation (0.481^*^, *p* < 0.05), information overload (0.647^**^, *p* < 0.01), social overload (0.362^**^, *p* < 0.01), self-efficacy (0.543^*^, *p* < 0.05), privacy anxiety (0.350^**^, *p* < 0.01), impression management (0.536^**^, *p* < 0.01), and intergenerational communication (0.342^**^, *p* < 0.01) were significantly associated with SMF. Therefore, eight variables were included in the multiple regression analysis.

To further analyze the degree of influence of each variable on SMF behavior, negative comparison, interaction dysregulation, information overload, social overload, self-efficacy, privacy anxiety, and impression management interaction dysregulation were set as independent variables and SMF behavior was set as the dependent variable, and multiple linear regression was conducted using SPSS, controlling for demographic variables, including gender, age, and income. In the theoretical construction of independent variable selection, there were deep theoretical differences in three aspects of relational stress, overload, and personal traits, so three layers of independent variables were put into the regression analysis. Finally, three regression models were obtained and the significant differences of the three models were similar. Through Table 4, we chose model three (adjusted *R*^2^ = 0.327, *p* < 0.001), which had the highest explanatory power, as the main basis for testing the hypotheses. The empirical data showed that the effects of privacy anxiety were not significant in the model and hypotheses H6 did not hold. Negative comparison (β = 0.227, *p* < 0.001) was significantly and positively related to SMF behavior and hypothesis H1 held. Interaction dysregulation (β = 0.221, *p* < 0.005) was significantly and positively related to SMF behavior and hypothesis H2 held. Information overload (β = 0.349, *p* < 0.005) was significantly and positively related to SMF behavior and hypothesis H3 held. Social overload (β = 0.196, *p* < 0.005) was significantly and positively related to SMF behavior and hypothesis H4 held. Self-efficacy (β = 0.283, *p* < 0.005) was significantly and positively related to SMF behavior, empirical data results and hypothesis H5 reversed. Impression management (β = 0.303, *p* < 0.005) was significantly and positively associated with SMF behavior and hypothesis H7 held. Intergenerational communication (β = 0.212, *p* < 0.000) was significantly and positively correlated with SMF behavior and hypothesis H8 held. The specific regression analysis table is shown in Table 4.

**Table 4:** Regression analysis table.

**Dependent variable**	**Social media fatigue behavior**								
**Predictive variables**	**Model one**			**Model two**			**Model three**		
**Control variable**	**β **	**t**	***P***	**β**	**t**	***P***	**β**	**t**	***P***
Gender	0.541	0.677	0.459	0.016	0.514	0.251	0.415	0.656	0.536
Age	0.134	4.322	0.115	0.324	4.601	0.191	0.540	2.842	0.317
Education level	0.131	5.305	0.158	0.238	2.452	0.638	0.157	6.375	0.045
income	0.351	4.654	0.621	0.523	3.145	0.728	0.524	5.093	0.551
**Independent variable**
Negative comparison	0.351	3.141	0.004	0.345	5.211	0.002	0.227	3.014	0.000
Interaction dysregulation	0.237	3.542	0.007	0.394	6.125	0.006	0.374	4.204	0.004
Information overload	0.417	2.943	0.003	0.562	4.410	0.002	0.349	2.036	0.003
Social overload	0.794	3.908	0.005	0.443	5.153	0.006	0.196	1.502	0.004
Self-efficacy	0.408	2.954	0.005	0.511	3.451	0.004	0.283	1.135	0.002
Privacy anxiety	0.572	3.245	0.182	0.603	8.505	0.304	0.237	3.156	0.426
impression management	0.205	2.852	0.008	0.431	6.236	0.102	0.303	2.632	0.002
Intergenerational Communication	0.391	4.923	0.005	0.259	5.445	0.002	0.212	5.320	0.000
*R* ^2^	0.251			0.277			0.362		
Adjusted *R*^2^	0.207			0.219			0.327		
*F*-value	20.644			18.019			10.241		
Significant F	0.002			0.001			0.003		

### Model Modification

**Figure 2:**
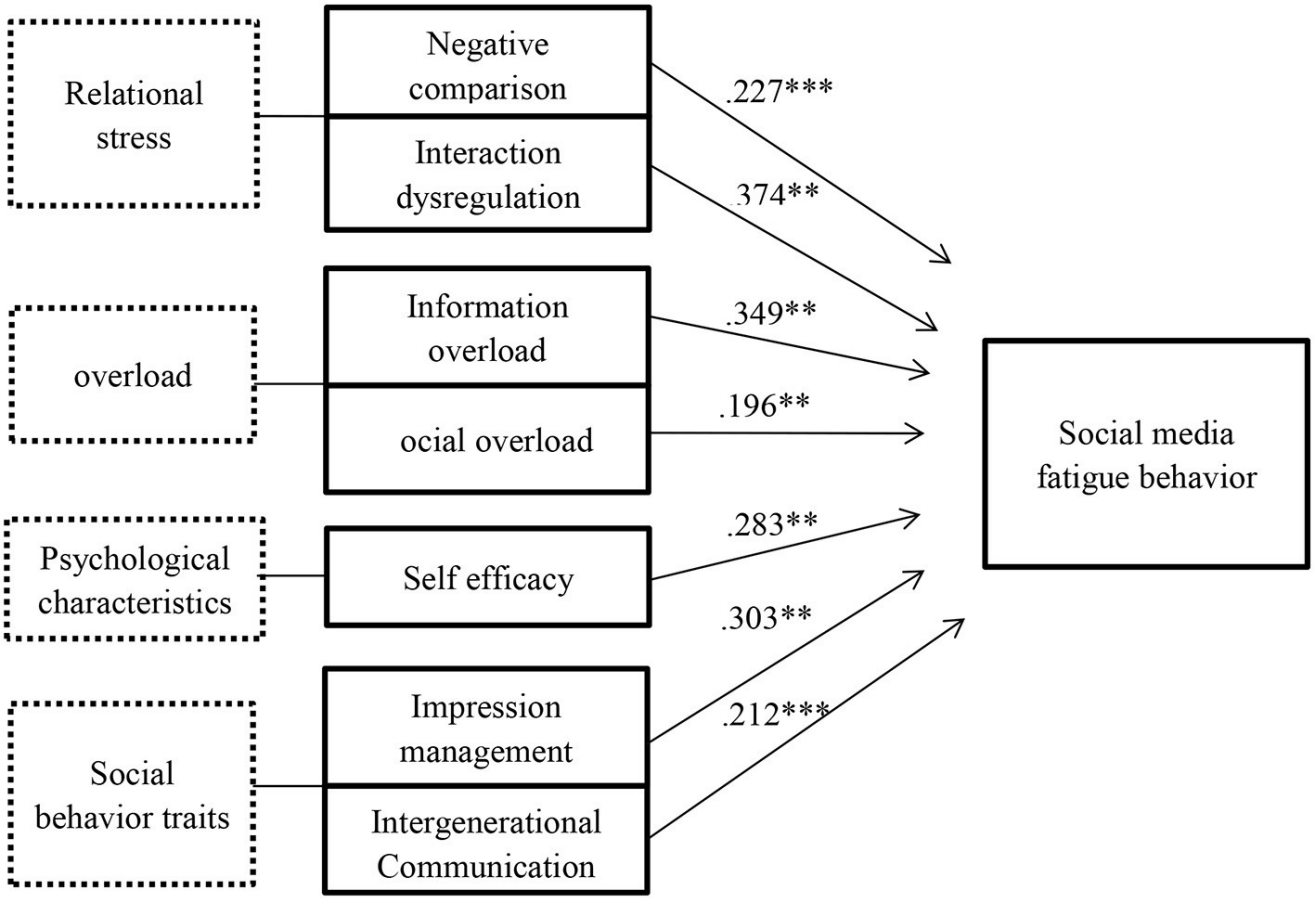
Model modification chart. **p < 0.01, ***p < 0.001.

From the results of the above analysis, it can be seen that six of the original hypotheses are valid, one hypothesis was not valid, and one hypothesis was contrary to the empirical results, which shows that negative comparison, interaction dysregulation, information overload, social overload, self-efficacy, impression management, intergenerational communication, and SMF behaviors were all significantly and positively influenced by the following revised model as shown in Figure 2.

### Research Results and Findings

This study explored the SMF behavior of youth groups from four aspects namely, relational stress, overload, personal-psychological traits, and social-behavioral traits. Furthermore, this study enriched relevant new variable conditions and further expanded the model of factors influencing SMF behavior of young people. Firstly, the empirical data showed that in terms of relational stress, negative comparison and interaction dysregulation have a significant positive effect on SMF behavior of youth groups. Meaning, information overload and social overload have a significant positive effect on social fatigue behavior, which is consistent with the findings of previous studies. In the section on personal psychological traits, self-efficacy and privacy anxiety were added to the factors influencing SMF behavior, and it was proved that privacy anxiety had no significant positive effect on SMF behavior in the youth group. Furthermore, this aspect also tested the conjecture of related scholars on the variable of self-efficacy, and the results of the study could provide reference and guidance for other subsequent studies of scholars. In addition, individual impression management and intergenerational communication were added to the model for validation, and the data showed a positive effect on SMF behavior. This enriched the variable conditions in the model and made the study more complete, and, to a certain extent, promoted the research process of this topic.

## Research Conclusion and Discussion

### Research Conclusion

The following is the specific analysis of each variable in this study based on the results of the empirical data.

In terms of relational stress, negative comparison and interaction dysregulation positively affect SMF. Some researchers have found that Qzone uses positively affects negative social comparison, and negative social comparison positively affects the depression of users (Niu et al., 2018). In turn, the author proposed the research hypothesis that negative social comparison positively affects SMF, and the empirical data also proved the objectivity of this hypothesis. This suggested that negative social comparisons brought about by social media are a common phenomenon among youth groups and that upward social comparisons made by young people with positive self-presenting others during social media use positively predict their SMF. Youth social media users may tend to respond to the stress of social comparison with negative emotions (Lim and Yang, 2015); thus, SMF may be a coping measure for youth in such stressful situations. However, in our interviews with some respondents, we found that young people with high self-esteem generally did not agree that people around them are better off than they are, and it is possible that they intentionally concealed their true thoughts since the questionnaire was anonymous and would reflect a more realistic inner situation. Thus, we believe that the relational pressure of negative social comparison cannot be ignored. A plausible explanation is that young people reduce uncertainty in their self-assessment by comparing themselves to others during social media use in order to obtain information (Gilbert et al., 1995). However, since other social media users prefer to present their positive selves (Reinecke et al., 2017), and to best represent their ideal selves in a way that describes themselves, youth social media users may develop negative emotions during this social comparison (Rosenberg and Egbert, 2011). Therefore, this study suggested that negative social comparison tendencies are significantly associated with negative experiences and fatigue during youth social media use.

Through the empirical data, we have seen that interaction dysregulation contributed the most to the model. In fact, social media contains complex social relations and social capital. SMF behavior cannot be simply regarded as the weariness of the individual toward media technology. The deeper sense of fatigue comes from the relational pressure brought by social media (Bane et al., 2010). Among young social media users, the interaction imbalance is mainly reflected in the weakening of individual social interaction and self-immersion out of control. More and more young people feel the pressure and ineffectiveness of social activities. Most of them, as independent individuals, will feel complex interpersonal pressure when they face social chat. Through the interview, we also found that many young people think that they have no friends. In the face of cold media technology in social media, only relying on online communication will make their friends in life go away. In addition, self-immersion out of control also has an important impact on the SMF of young people. The self-control ability of young people is relatively poor, and the entertainment and interaction of social media easily make them indulge in it. When users wake up from the immersion state and perceive the waste of time and energy, they are likely to feel guilty and thus reduce their interest in using social media (Ravindran et al., 2014b).

(2) In terms of information overload and social overload, empirical data showed that information overload and social overload positively affect social fatigue behavior, which can also confirm previous theoretical results. Several studies have proved that the energy of people in processing information is limited. Some interviewees said that with regard to information overload, including a large number of useless advertisements and excessive software services, users will feel tired when the quality of the system is low. Through participation and observation, we also find that social media plays an important role in the daily life of young people, and even becomes the only channel for people to obtain information and socialize. People put a lot of energy into it and must feel the huge pressure of information overload and relationship maintenance. At the same time, some respondents said that when the information is too complicated, they will pay more attention to check their social media accounts, hoping to obtain key information. However, there will be more invalid information and similar information usually, further aggravating their fear of missing key information. They will pay more energy and time costs, thus feeling overwhelmed and causing bad emotions. In the face of this situation, many young people take an indifferent attitude to social information. The pressure of information overload and social overload makes individuals have the behavior of staying away from social media.

(3) The empirical data showed that self-efficacy positively influences social fatigue behavior, a finding that is contrary to the research hypothesis. In the hypothesis formulation process, self-efficacy theory is built on the level of confidence that the youth group has the skills and accomplishes a certain task. In fact, the acquisition of individual self-efficacy is nonetheless far more complex than we think, and individual self-efficacy includes several pathways through which self-efficacy is acquired through first-hand learned experiences, alternative learned experiences, social feedback, and positive physical and emotional states (Bandura, 1982). Nowadays, more and more people rely on social media, especially young people who are “Internet natives” and rely more on the Internet and social media for information. Most of them have the ability to use information tools skillfully. In our initial hypothesis, we assumed that young people would be unable to master social media skills and thus develop SMF behavior, but such young people represented only a very small minority of the sample. The sample data showed that the younger cohort generally had a high level of self-efficacy, and during the interviews, we also found that respondents with a high level of social media self-efficacy were likely to use social media to a higher degree because they felt capable of using this form of media. As a result, they may experience high levels of SMF due to increased usage. This means that as social media users become more self-efficacious, their experiences will encourage further social media use (Bandura, 1982). Individuals with low self-efficacy are less likely to perform related behaviors in the future, which also means that users with low self-efficacy are less motivated to engage in social media-related behaviors and activities than those with high self-efficacy. Also, individuals with higher self-efficacy have higher self-esteem (Compeau and Higgins, 1995). Through interviews, we found that the parent groups with high self-efficacy would carefully consider the interest and accuracy of their published content before updating their published articles. Most of them would pursue the quality of published content, so they would become more cautious in publishing, and SMF would occur over time.

In terms of privacy anxiety of young people, it has no significant positive effect on SMF. In various research of scholars, privacy anxiety positively affects SMF behavior. Because social media as a tool for interaction between friends, information exchange is established within a certain boundary. However, if this boundary is broken, it will lead to individual privacy concerns and further results in SMF behavior (Bright et al., 2015). Nonetheless, the sample in this study focused on young social media users aged 18–29, most of whom grow up in the environment of rapid development of network society and have good confidence in the network environment. Through an interview, some interviewees said that the social media technology platform also has good privacy protection schemes, such as publishing information content group visible, limiting the visible range of information publishing, comment protection, and strengthening blacklist, among other methods. They also fully trust the network security technology. For the anonymity and openness of social media, they say that even if their privacy is exposed, it will not cause great losses (Wang et al., 2006).

(4) In social behavior traits, individual impression management and interaction disorder have a significant positive impact on social fatigue behavior. In social media, the pictures and words we publish every day are an important self-presentation management strategy (Pearson, 2010). In the view of Goffman, performance is everywhere. In the real world, we shape the social identity of an individual through the front stage display and self-presentation. The front stage consists of “appearance” and “manner” stimuli. We expect certain consistency between appearance and behavior to shape our role, which can make social interaction continue (Goffman, 1959). Through the interview, we found that young people in social media individuals will also maintain the reality of self-presentation. In order to show their good image, they will generally show their positive image on social media, with the intention of weakening their own shortcomings through good impression management. At the same time, we also found that the self-image presented by some young people in social media is quite different from that in reality. They may present a completely different image because of their work and social needs. Once they “disguise” themselves, they may be another new identity. This identity may be different from the real self, and this “performance style” self-presentation, needs to be shaped by individual impression management. However, this kind of impression management behavior will make young people easily feel tired in the process of social media self-presentation. They will no longer update their information status and shape their own image with the help of social media platforms, resulting in SMF behavior.

Empirical data showed that intergenerational communication positively affects SMF among youth. The Internet has exacerbated the digital divide between offspring and fathers. The differences in online expression, information selection, and media use habits have become important causes of intergenerational conflicts. Youth groups often take measures such as setting groups and blocking friends to avoid the excessive interference of their elders in their online social activities. Although this approach can reduce conflicts to a certain extent, the related settings are more cumbersome, which will affect the experience and comfort of social media use by the offspring, Moreover, there is a risk of being detected by the perception of elder, which will easily make users anxious and worried. During our interviews, many young people said that the development of Internet media has made them form new values, and the communication between them and their parents will be more separated, forming an irreconcilable digital divide. The complexity and variability of the online environment make parents more worried about the online social situation of their children, which can lead to over-intervention (Jiang and Gong, 2016). Young people are too lazy to explain to their parents, resulting in a growing intergenerational divide between them. This circumstance further affects family relationships and directly affects their emotions about social media use, leading to SMF.

### Research Discussion

According to the results of the study, among the seven factors influencing social fatigue behavior, which are interaction dysfunction, information overload, and impression management, the last one made the highest contribution in the model. This result confirmed the influencing factors such as relational stress and platform information overload mentioned in many studies. Several studies have proved that people avoid coping in the face of massive amounts of information and feel tired in the face of complex and diverse online interpersonal relationships. The empirical results of this study further suggested that interpersonal stress may not be the main source of SMF. In-depth interviews with selected youth groups revealed that the problem with social media for youth may not be that people are overwhelmed by daily chats, but that the functionality of social media has changed. Many interviewees mentioned the relationship between social media and work, the increasing number of group chats, and the fact that most social media group chats are created for organizational and work purposes. In the original sense, social media is a field for private social activities. However, with the development of the social media era, the public sphere has gradually invaded the private sphere wherein work matters are increasingly colonized into private space through social media (Endeshaw, 2004). Both interaction dissonance and information overload directly reflected this problem. Because daily social activities are linked to capital, the youth group may not have reasonable control over their time use, even if they feel uncomfortable. Respondents also reported that such SMF is not only aggravated, but also accompanied by various anxieties such as “I can't miss important information,” and the more anxious they are, the more fatigue they will experience, thus creating a vicious circle. This relational pressure and overloaded media environment are overwhelming for the youth. Respondents also reported that it takes a lot of self-discipline and even some cost when they want to control their social media usage time to combat this pressure that has invaded their private lives. Therefore, in our observations, we found that it is important to examine the impact of this relational pressure on social fatigue behavior by focusing not only on online interpersonal pressure but also on the relational pressure between people and technology and capital. Behind the SMF behavior is the alienation that is brought by social acceleration to the youth group in the whole range of space, time, thought, experience, interaction, and behavior (Lee et al., 2013).

Social behavioral traits are also important influencing factors in SMF behavior, and the maintenance of self-image and selective presentation of individuals in social media interaction studies is a topic that cannot be ignored (Laghi et al., 2011). In this study, online impression management has been shown as an important influencing factor of the SMF behavior of young people. When individuals present themselves in different scenarios, they tend to highlight the part of their role traits that are adapted to the current situation and hide other traits. Evidently, how to present oneself and manage the self-image of an individual in a virtual environment is still an important part of online social interaction. However, many respondents said that although online identity building can create a good identity image, by showing videos, photos, and images on social media, the sense of self of an individual can be shown. This in turn has a role-building effect and makes it easier for people to idealize the person they are communicating with. Such behavior can also create an inauthentic social environment, where everyone wants to present the best side of themselves. Furthermore, when online relationships were transferred to offline relationships, many real images and those managed online do not match, increasing social risks and the negativity of social comparisons (Endeshaw, 2004). Over time, the youth group is tired of managing their online identity image and social fatigue is becoming stronger.

At the same time, respondents said that relationships in social media are equally divided into two different states, strong and weak, based on the proximity of interpersonal relationships. The so-called strong ties are a relationship state with long interaction time, strong emotional color, forming a certain intimacy, such as the connection with friends and relatives in reality, which is reflected in the weak ties on the contrary (Granovetter, 1973). These acquaintances are the groups of good friends, workmates, lovers, and family members in the chain of “strong ties.” However, within these different strong ties, there are still significant differences. Specifically, in online communication with acquaintances, workmates, lovers, and other strong ties are grouped at the same level. Moreover, there is no communication difference at the same level, communication is more equal, and the other party does not pose pressure on their interaction behavior. If acquaintances, friends, and colleagues are strong ties groups with the communicator at the same level, then parents, elders, mentors, and leaders, re actually in a non-reciprocal communication relationship with the communicator, i.e., there is a hierarchical difference of “superior-subordinate” (Bian, 1994). Furthermore, in the interpersonal network, in addition to the horizontal “closeness” difference, there is also another vertical hierarchy, which sometimes causes intergenerational miscommunication or a huge digital information gap. This, in turn, interferes with the daily life behavior of the youth group and puts more pressure on their social media behaviors causing greater pressure. Some respondents also said that when public affairs invade the social life of individuals, many online friends and workmates are not familiar with each other and it is a “weak tie” (Granovetter, 1983), for which the posting of life status may cause privacy disclosure and unnecessary troubles. This leads to SMF, which eventually makes the youth group avoid social media.

## Strengths

The strengths and theoretical contributions of this study are elaborated. This is a very interesting topic, young people are active in social networks and are an important part of social media users, but various reasons have caused SMF behavior in the youth group in recent years. Thus, we have obtained some important influencing factors through empirical research, some findings can verify previous studies, some findings are innovative. The study expanded the research related to the field of people and technology. For example, this study expanded the study of SMF behavior in youth groups, enriched new influencing factors, and explained them with appropriate empirical materials and academic theories. In addition, previous studies on social fatigue have mostly been conducted from perceptions and social relationships in psychology, but this study also enriched the influence of relational stress and personality psychology and behavioral traits on SMF behavior, with a more comprehensive perspective. New variables which were not focused on in previous studies were added, such as the effects of negative comparison, self-efficacy, and intergenerational communication on SMF behaviors. In terms of research methodology, this study combined quantitative and qualitative approaches, which is more scientific and objective and led to more consistent conclusions, presenting objectively the relationship between fatigue behaviors of youth groups and their influencing factors with precise data and interpretation of empirical materials. The empirical results obtained were more standardized and reliable, which enriched the research content of social media user behavior and expands the research ideas in this field.

From a practical point of view, the empirical research results could help social media operators understand the formation mechanism of SMF behavior, to effectively avoid the problems of declining activity and user loss caused by SMF behavior. The middle-aged and elderly population is concerned about the usefulness and ease of use of technology platforms, but the youth group is not. Therefore, the usefulness and ease of use of the media platform should not be deliberately emphasized, and different service functions should be set according to the different needs of the audience. In order to avoid information overload, developers and operators should provide information filtering and information management functions to improve the efficiency of the use of social media of users, help users better manage their own information networks and social relationship networks, better use social media to carry out related services, strengthen the user experience, and effectively manage SMF behavior according to the interactions of influencing factors.

## Limitations

This study also has some limitations. First of all, this study uses a convenient sample, which involved people aged 18–35. Most of the participants are young social media users, so the results of this study cannot be extended to a wider range of the whole. The age difference of users was small, which cannot compare the differences of SMF behavior of users of different ages, which to a certain extent narrowed the scope of the study and affected the comprehensiveness of the data. In future research, we should increase the sample size to avoid data concentration and involving different age groups. In addition, this study did not limit the types of social media platforms, because different social media technology platforms also have differences, and the SMF behavior of users will also have differences. In the future, we can compare the differences in the SMF levels of users on different types of social media platforms. At the same time, because social media has largely blurred the boundaries between the private field and the work field (Bucher et al., 2013), future research can also explore the potential causes and consequences of SMF in different life scenarios.

The model of this study was based on the discussion of personal psychological and behavioral characteristics. Through the collation of relevant influencing factors of previous studies and some interview empirical materials, we could obtain the influencing factors of SMF behavior of youth groups as independent variables, but it may not be limited to these conditions. The later research should expand the exploration field and add the introduction of new variables to improve the corresponding research methods Theoretical model. In future research, it is necessary to increase the strength of interviews, as much as possible multi-dimensional analysis and research, to make this research more universal and reasonable. The grasp of SMF behavior is helpful to promote the sustainable development of social media platforms in the information society and promote the harmonious progress of human society. Therefore, the research on SMF behavior needs to be further explored.

## Data Availability Statement

The original contributions presented in the study are included in the article/supplementary material, further inquiries can be directed to the corresponding author/s.

## Ethics Statement

The studies involving human participants were reviewed and approved by Shenzhen University Ethics Committee. The patients/participants provided their written informed consent to participate in this study. Written informed consent was obtained from the individual(s) for the publication of any potentially identifiable images or data included in this article.

## Author Contributions

YL is responsible for the overall research design and thesis writing. JH is responsible for the collation of the questionnaire and data guidance. All authors in the research team has contributed to the thesis.

## Funding

This study is a phased achievement of Guangdong Province philosophy and social science planning project Research on prevention strategies of youth's addiction to online social games (project number: GD20CMK06).

## Conflict of Interest

The authors declare that the research was conducted in the absence of any commercial or financial relationships that could be construed as a potential conflict of interest.

## Publisher's Note

All claims expressed in this article are solely those of the authors and do not necessarily represent those of their affiliated organizations, or those of the publisher, the editors and the reviewers. Any product that may be evaluated in this article, or claim that may be made by its manufacturer, is not guaranteed or endorsed by the publisher.
